# Chloroquine-Resistant Haplotype *Plasmodium falciparum* Parasites, Haiti

**DOI:** 10.3201/eid1505.081063

**Published:** 2009-05

**Authors:** Berlin L. Londono, Thomas P. Eisele, Joseph Keating, Adam Bennett, Chandon Chattopadhyay, Gaetan Heyliger, Brian Mack, Ian Rawson, Jean-Francois Vely, Olbeg Désinor, Donald J. Krogstad

**Affiliations:** Tulane University, New Orleans, Louisiana, USA (B.L. Londono, T.P. Eisele, J. Keating, A. Bennett, B. Mack, D.J. Krogstad); University of Pamplona, Pamplona, Colombia (B.L. Londono); Hôpital Albert Schweitzer, Deschapelles, Haiti (C. Chattopadhyay, G. Heyliger, I. Rawson); Swiss Tropical Institute, Basel, Switzerland (C. Chattopadhyay); Ministry of Health, Port-au-Prince, Haiti (J.-F. Vely); US Agency for International Development, Port-au-Prince (O. Désinor)

**Keywords:** Malaria, parasites, Plasmodium falciparum, Haiti, chloroquine, P. falciparum chloroquine resistance transporter, haplotypes, research

## Abstract

*Plasmodium falciparum* parasites have been endemic to Haiti for >40 years without evidence of chloroquine (CQ) resistance. In 2006 and 2007, we obtained blood smears for rapid diagnostic tests (RDTs) and filter paper blots of blood from 821 persons by passive and active case detection. *P. falciparum* infections diagnosed for 79 persons by blood smear or RDT were confirmed by PCR for the small subunit rRNA gene of *P. falciparum.* Amplification of the *P. falciparum* CQ resistance transporter (*pfcrt*) gene yielded 10 samples with amplicons resistant to cleavage by *Apo*I. A total of 5 of 9 samples had threonine at position 76 of *pfcrt*, which is consistent with CQ resistance (haplotypes at positions 72–76 were CVIET [n = 4] and CVMNT [n = 1]); 4 had only the wild-type haplotype associated with CQ susceptibility (CVMNK). These results indicate that CQ-resistant haplotype *P. falciparum* malaria parasites are present in Haiti.

## Abstract 2

Chloroquine resistance is now present in this country.

The island of Hispaniola is the only area in the Caribbean Sea where *Plasmodium falciparum* malaria is endemic (*1*). It has been reported that up to 75% of the population of Haiti lives in malarious areas, especially at altitudes <300 m above sea level (*2**,**3*). *P*. *falciparum* is the only malaria parasite species that causes malaria in Haiti. The last confirmed endogenous case of *P*. *vivax* malaria was in 1983 (*4*); 6 cases of *P*. *malariae* malaria were reported recently in Haitian refugees in Jamaica (*5*).

Haiti has been a remarkable outlier as a country in which *P*. *falciparum* malaria is endemic without evidence of chloroquine (CQ) resistance (*3**,**6**–**8*). Even though Haiti has had no comprehensive national malaria control program for 20 years (*9*), several reports have found no evidence of CQ resistance in Haiti (*3**,**6**–**8*). Those reports are consistent with the conclusions of domestic and international health agencies, which recommend CQ for the prevention of malaria in Haiti and the treatment of patients with malaria acquired in Haiti (*8**–**10*).

Accordingly, the original objectives of this research focused not on CQ resistance but on quantifying *P*. *falciparum* infection, including the heterogeneity and multiplicity of infection, and on identifying factors associated with low-intensity transmission in the Artibonite Valley of Haiti (*11**,**12*). We describe secondary analyses of blood samples for CQ-resistant *P*. *falciparum* haplotypes from samples collected in 2006 and 2007 that previously tested positive (*11**–**13*).

## Materials and Methods

### Ethical Approval

The protocols for these studies were reviewed and approved by the Institutional Review Boards of Tulane University and the Hôpital Albert Schweitzer (Deschapelles, Haiti). All samples were collected after obtaining informed consent.

### Study Site

Studies were performed in the low-lying Artibonite Valley. The valley has abundant rainfall and is heavily farmed; 80% is irrigated for the cultivation of rice and other crops. The major peak in malaria cases (>99% caused by *P*. *falciparum*) (*11**,**14**–**16*) is during November–January (*11**,**12**,**17*). The population of the Artibonite Valley relies primarily on subsistence farming and informal trade (barter) for income. This population is poor; only 18% of households have electricity and just 12% have piped water (*12*). As a result, members of the population rarely travel outside the study area, and international travel to other malaria-endemic countries is uncommon. The primary malaria control activities currently being implemented include improvement of microscopy at Hôpital Albert Schweitzer, a facility that is supported by the Global Fund (www.theglobalfund.org/en/worldmalariaday/2007) and vector control (*10*).

Hôpital Albert Schweitzer was the base of operations for the household surveys, passive case detection, and laboratory studies (i.e., thick and thin blood smears, antigen testing by using rapid diagnostic tests [RDTs], clinical examinations, and clinical and laboratory follow-up of patients). This hospital provides comprehensive inpatient care at its 100-bed facility and delivers preventive and primary health services to a population of 300,000 through a network of health centers, dispensaries, and workers in the community. Data from Hôpital Albert Schweitzer indicate that malaria transmission in this area of Haiti varies annually according to rainfall. For example, 157 of 2,739 suspected cases were confirmed by microscopy and treated with CQ in 2005 (smear positivity rate 5.7%), and only 29 of 1,307 suspected cases were confirmed and treated in 2006 (smear positivity rate 2.2%). The prevalence of *P*. *falciparum* infection in this area of Haiti is estimated to be 3.1% (*13*).

### Household Survey in 2006 (Active Case Detection)

A 2-stage cluster design, in which probability was proportional to cluster size, was used to generate a sample of 200 households within the study area, as described elsewhere (*11**,**12*). Thick and thin blood films and 4 blots of blood on filter paper for PCR were collected from 714 persons >1 month of age within selected households. All smear-positive case-patients were treated with CQ.

### Passive Case Detection in 2006 and 2007

#### Data for 2006

Four blots of blood on filter paper (each containing 50 μL) and axillary temperatures were obtained from 55 persons (age range 11–80 years) with clinically suspected cases of malaria who came to Hôpital Albert Schweitzer during December 2006. All 55 samples were tested for *P*. *falciparum* infection by using PCR.

#### Data for 2007

As part of pilot studies of a passive case detection system to identify households with malaria, 4 blots of blood on filter paper and axillary temperatures were obtained before treatment with CQ. Forty-seven smear-positive persons 2–84 years of age were seen and treated at Hôpital Albert Schweitzer or a nearby satellite clinic in Liancourt from November 5 through December 3, 2007. A data collection team was sent to households of 45 positive case-patients within 3 days for blood sample collection from all household residents >1 month of age. Thick and thin blood films, a drop of blood for an RDT (OptiMAL-IT; DiaMed AG, Cressier sur Morat, Switzerland), 4 blots of blood on filter paper, and axillary temperatures were obtained from 249 household members 2–85 years of age. Five of these persons (age range 5–37 years) had positive results for *P*. *falciparum* by RDT and were treated with CQ. Fifty-two samples from persons who had either a positive smear at Hôpital Albert Schweitzer or a positive RDT result at home were then examined for *P*. *falciparum* infection by using PCR.

### Diagnosis of Malaria by Blood Smear or RDT and Species-Specific PCR for *P. falciparum* Small Subunit rRNA Gene

Thick and thin Giemsa-stained blood smears were examined for malaria parasites at Hôpital Albert Schweitzer by trained laboratory technologists by using standard methods (*18**,**19*). Filter paper blots were transported from Haiti to New Orleans where parasite DNA was extracted (*20**,**21*), and microscopy results were confirmed by using a PCR for the *P*. *falciparum* small subunit (SSU) rRNA gene (*22*). DNA was extracted from filter paper blots by using the Charge Switch Forensic DNA Purification Kit (catalog no. CS 11200; Invitrogen, Carlsbad, CA, USA) according to the manufacturer’s instructions. This extraction yielded 150 μL of DNA in buffer (10 mmol/L Tris, pH 8.5, 1 mmol/L EDTA) from each specimen.

PCR for the *P*. *falciparum* SSU rRNA gene used a *P*. *falciparum*–specific forward primer (which hybridizes only with *P*. *falciparum* DNA) and a genus-specific reverse primer (which hybridizes with DNA from all 4 *Plasmodium* spp. that infect humans: *P*. *falciparum*, *P*. *vivax*, *P*. *ovale*, and *P*. *malariae*) (*22*) (Table 1). To perform this PCR, 4 μL of DNA extracted from filter paper blots was added to 19 μL of PCR master mixture (Promega, Madison, WI, USA) and 1 μL of each primer. Parasite DNA was amplified after an initial denaturation at 95°C for 15 min; 43 cycles of denaturation at 95°C for 45 s and annealing at 60°C for 90 sec; and a final extension at 72°C for 5 min in an i-Q thermocycler (Bio-Rad, Hercules, CA, USA). Positive controls for these assays contained DNA from in vitro culture of the Haiti I/CDC strain of *P*. *falciparum* (*26*). Resulting amplicons were visualized by electrophoresis on 1% agarose gels stained with ethidium bromide (*27**,**28*). Amplicon sizes were estimated by using a 100–600-bp DNA ladder (catalog no. 15628–019; Invitrogen).

**Table 1:** Primers used to amplify *Plasmodium falciparum* DNA during study in Haiti*

Primers (5′ → 3′)	Amplicon, bp	Tm, °C	Reference
Primers for *P. falciparum* species-specific SSU rRNA gene	276		(*22*)
Forward: AACAGACGGGTAGTCATGATTGAG		56.5	
Reverse: GTATCTGATCGTCTTCACTCCC		54.5	
Primers for single-step *pfcrt* gene PCR	170		(*23*)
Forward: TgTgCTCATgTGTTTAAACTT		50.6	
Reverse: AATAAAgTTgTgAgTTTCggA		49.8	
Primers for nested (2-step) *pfcrt* gene	573		(*24*,*25*)
First round of amplification			
Forward (CRTP1): CCGTTAATAATAAATACACGCAG		49.9	
Reverse (CRTP2): CGGATGTTACAAAACTATAGTTACC		51.5	
Second round of amplification			
Forward (CRTD1): TGTGCTCATGTGTTTAAACTT	134	50.6	
Reverse (CRTD2): CAAAACTATAGTTACCAATTTTG		46.1	

*Nucleotides in upper case letters were conserved in 100% of sequences at those positions, and nucleotides in lower case letters were conserved in most (e.g., >50%) sequences at those positions. Tm, melting (annealing) temperature; SSU, small subunit; *pfcrt*, *P. falciparum* chloroquine resistance transporter.

### Amplification of *P. falciparum* *pfcrt* Gene from Specimens Positive for *P. falciparum* SSU DNA

Two protocols were used to amplify the *P*. *falciparum* CQ resistance transporter (*pfcrt*) gene responsible for CQ resistance (*23**–**25*). The first protocol (single-step PCR) was used to screen 79 smear-positive and RDT-positive specimens that were positive for *P*. *falciparum* SSU DNA (*23*). In this assay, 4 μL of DNA extracted from filter paper blots was mixed with 21 μL of PCR master mixture (Promega) plus 2 μL of primers (Table 1) and amplified by an initial denaturation at 95°C for 7 min; 40 cycles of denaturation at 94°C for 30 sec, annealing at 57°C for 30 sec, and extension at 72°C for 30 sec; and a final extension at 72°C for 10 min in an i-Cycler thermocycler (Bio-Rad).

The second protocol (nested PCR) (*24**,**25*) was used to retest 58 specimens positive for SSU DNA that were negative in the single-step PCR for *pfcrt*. The nested PCR protocol used primers CRTP1 and CRTP2 for the first round of amplification and primers CRTD1 and CRTD2 for the second round (*24**,**25*). Samples in the first round were amplified by an initial denaturation at 94°C for 3 min; 45 cycles of denaturation at 94°C for 30 sec, annealing at 56°C for 30 sec, and extension at 60°C for 1 min; and a final extension at 60°C for 3 min. Samples in the second round were amplified by an initial denaturation at 95°C for 5 min; 30 cycles of denaturation at 92°C for 30 sec, annealing at 48°C for 30 sec, and extension at 65°C for sec; and a final extension at 65°C for 3 min (Table 1).

### Digestion of Amplicons from *pfcrt* with *Apo*I

For each sample positive for SSU DNA, an aliquot (10 μL) of the *pfcrt* gene PCR product was digested with 10 U of *Apo*I (New England Biolabs, Beverly, MA, USA) according to the manufacturer’s instructions. Briefly, 10 U of *Apo*I in 1× NE buffer 3 (100 mol/L NaCl, 50 mmol/L Tris-HCl, 10 mmol/L MgCl_2_, 1 mmol/L dithiothreitol) and bovine serum albumin (100 μg/μL) were incubated overnight with 10 μL of the PCR product at 50°C (*23**–**25*). DNA fragments from samples and positive and negative controls were resolved by electrophoresis on 3% agarose gels stained with ethidium bromide.

*Apo*I digests most wild-type *pfcrt* genes (with CVMNK haplotype sequences at positions 72–76) but not the CQ-resistant mutant gene (i.e., K76, not T76) (*23**–**25*). On the basis of a single-step PCR for *pfcrt,* which yields an amplicon of 170 bp, amplicons with a lysine at position 76 (K76) are digested into 2 fragments (98 bp and 72 bp). Amplicons from CQ-resistant parasites (i.e., parasites with CVIET and CVMNT sequences at positions 72–76) are not digested by *Apo*I, resulting in an unchanged amplicon of 170 bp. The nested PCR product is slightly smaller (134 bp vs. 170 bp). As with the single-step PCR, most amplicons from CQ-susceptible parasites are digested by *Apo*I (in this instance to 30-bp and 104-bp fragments); amplicons from CQ-resistant parasites are not digested (unchanged amplicons of 134 bp; *24,25*).

### Amplification, Cloning, and Sequencing of *pfcrt* Genes Not Digested by *Apo*I

Samples not digested by *Apo*I for which DNA was available (9 of 10) were reamplified under the conditions described above for nested *pfcrt* PCR, cloned into the pCRII-TOPO vector, and transfected into the TOP10 strain of *Escherichia coli* by using the TOPO TA Cloning Kit (Invitrogen) according to the manufacturer’s instructions (*29*,*30*). Cloned *pfcrt* amplicons were sequenced in both directions by using CRTD1 and CRTD2 primers at an automated DNA sequencing facility (Davis Sequencing, Davis, CA, USA). Data for >3 clones sequenced in both directions were compared by using the multiple sequence alignment function in Lasergene version 7.2 software (DNASTAR, Madison, WI, USA) (*31*,*32*).

## Results

We identified 79 *P*. *falciparum* infections in 821 persons by using PCR for the *P*. *falciparum* SSU rRNA gene (Table 2) (*23*–*25*). The 51 persons identified by passive case detection were thought to have malaria because their temperatures were >37.5°C. In contrast, only 9 (39%) of 23 infected persons identified by active case detection in the 2006 household survey had temperatures >37.5°C (*11*). The *pfcrt* gene was amplified from these 79 samples by using either single-step (n = 21) or nested PCR (n = 58). After digestion by *Apo*I, 10 samples did not yield the 100-bp and 34-bp fragments characteristic of the CQ-susceptible *pfcrt* gene (Figure). PCR-amplified *pfcrt* DNA from 9 of these samples (no DNA was available for the 10th sample) was cloned into the TOPO TA vector, transfected into the TOP10 strain of *E*. *coli*, grown on selective medium, and sequenced. Sequences from 5 of 9 samples had *pfcrt* haplotypes associated with CQ resistance (5/79 [6%]; 4 CVIET and 1 CVMNT); 4 of these 5 samples were mixed infections that also had CQ-susceptible haplotype sequences (CVMNK). The remaining 4 samples had only sequences associated with CQ susceptibility (CVMNK) (Table 2). Although CQ treatment failures have not been reported in Haiti, no follow-up information was available for the 5 persons with CQ-resistant haplotype parasites.

**Table 2:** Samples from household surveys (active case detection) and hospital outpatients (passive case detection) tested by small subunit PCR for *Plasmodium falciparum*, by year of collection, Haiti

Characteristic	2006	2007	Total
Samples from household surveys, no. positive/no. tested (%)	23/714 (3.2)	5/5 (100)	28/719 (4)
Samples from hospital outpatients, no. positive/no. tested (%)	9/55 (16)	42/47 (89)	51/102 (50)
Total, no. positive/no. tested (%)	32/769 (4.2)	47/52 (90.4)	79/821 (9.6)
Molecular studies			
Resistance to *Apo*I digestion, no. positive/no. tested (%)	6/32 (19)	4/47 (9)	10/79 (13)
Haplotype, no. samples			
CVIET	4	0	4
CVMNT	0	1	1
CVMNK	2	3	5

**Figure:**
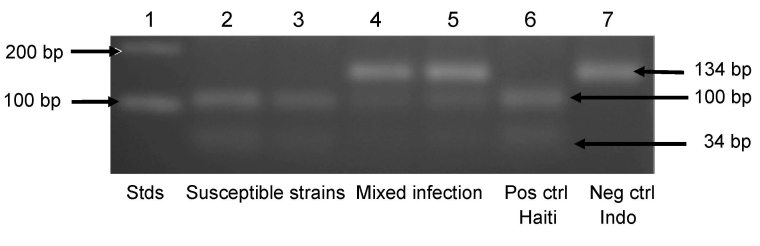
Agarose gel electrophoresis of amplicons for the *Plasmodium falciparum* chloroquine (CQ) resistance transporter gene digested with *Apo*I. Lane 1, DNA molecular mass standards (Stds) (Invitrogen, Carlsbad, CA, USA); lanes 2 and 3, amplicons susceptible to cleavage by *Apo*I, showing 2 fragments of 100 and 34 bp, consistent with infection by only CQ-susceptible haplotype parasites; lanes 4 and 5, amplicons partially resistant to cleavage by *Apo*I, showing 3 fragments of 134, 100, and 34 bp, consistent with mixed infections by CQ-resistant and CQ-susceptible haplotype parasites; lane 6, positive control (Pos ctrl), amplicon from CQ-susceptible Haiti I/CDC strain (*26*), showing 2 fragments of 100 and 34 bp; lane 7, negative control (Neg ctrl), amplicon from CQ-resistant Indochina (Indo) I/CDC strain (*33*), showing 1 fragment of 134 bp.

## Discussion

For as long as CQ has been available, *P*. *falciparum* has been endemic to Haiti without evidence of CQ resistance. During the past 20 years, several reports have noted the continued susceptibility of *P*. *falciparum* to CQ in Haiti (*3**,**6**–**9*), although Haiti had no comprehensive national malaria control program (*10*). Our results indicate that CQ-resistant haplotype *P*. *falciparum* parasites are now present in Haiti.

Our study has several limitations. First, because data on CQ-resistant parasites were not obtained from probability-based sampling, we were unable to estimate the potential effect and distribution of CQ resistance in the general population of Haiti. We can only report the presence of CQ-resistant haplotype parasite sequences in this area of Haiti. Second, we have not performed in vivo studies of treatment with CQ in Haiti to confirm molecular evidence for CQ resistance. Lastly, because these studies were based on results of filter paper blots, we have not yet been able to examine live *P*. *falciparum* parasites from the study area to test the effects of CQ on those parasites in vitro.

Beginning with studies of Djimde et al. (*24*) and Fidock et al (*34*), several studies have established a cause-and-effect relationship between the K76T point mutation (lysine → threonine at position 76 of *pfcrt*) and CQ resistance (*23**,**25**,**35*). In addition, studies in Southeast Asia, South America, and Africa have shown that persons who do not clear *P*. *falciparum* parasitemias after treatment with CQ have parasites that contain the K76T point mutation (*36**–**39*). Thus, *P*. *falciparum* parasites with CQ-resistant haplotypes that we identified in Haiti are likely to reduce the efficacy of CQ in Haiti as they have in sub-Saharan Africa, South America, and Southeast Asia (*36**–**39*).

Because the frequency of CQ-resistant *P*. *falciparum* in Haiti may be low, we suggest continuing CQ chemoprophylaxis for travelers to Haiti as currently recommended (*14**,**40*). We also suggest continuing to treat patients with uncomplicated *P*. *falciparum* infections acquired in Haiti with CQ in the absence of CQ chemoprophylaxis. However, if the presence of CQ-resistant *P*. *falciparum* in Haiti is confirmed by in vivo studies of resistance in humans or in vitro studies of parasite resistance to CQ, tourists and other nonimmune persons who acquire *P*. *falciparum* infections in Haiti or after travel to Haiti despite CQ chemoprophylaxis should be treated with alternative antimalarial drugs (mefloquine, atovaquone plus proguanil [Malarone], or sulfadoxine-pyrimethamine [Fansidar]), as they would be treated in other regions of the world where CQ resistance is present.

There are at least 2 potential explanations for CQ-resistant haplotype parasites in Haiti. First, CQ-resistant parasites may have been imported into Haiti by persons who acquired CQ-resistant *P*. *falciparum* in areas with established resistance, such as South America, sub-Saharan Africa, or Southeast Asia, where CVMNT and CVIET haplotypes circulate on a regular basis. Although this hypothesis could explain the presence of CVIET haplotype parasites in Haiti, it would require an initial importation by persons with greater financial resources than the residents of the Artibonite Valley. Second, CQ-resistant CVMNT haplotype parasites may have arisen by a single point mutation at position 76 in the *pfcrt* gene among naturally infected persons in Haiti, a mutation that could convert the predominant CQ-susceptible CVMNK haplotype to a CQ-resistant CVMNT haplotype. Defining the origin of these haplotypes will require additional sequencing within the *pfcrt* gene (beyond the 134-bp amplicon we studied) and at other loci.

At the Hôpital Albert Schweitzer and across Haiti, no clinical failures with CQ have been reported, and fatal cases of malaria are extremely rare. However, because CQ remains the first-line antimalarial drug in Haiti, selection for CQ-resistant parasites will continue and is likely to decrease the efficacy of CQ. Therefore, we suggest that now would be an opportune time to eliminate malaria from the island of Hispaniola before CQ resistance becomes broadly established, renders CQ ineffective, and makes elimination more much difficult. A commitment to eliminate malaria on Hispaniola would also provide an opportunity to test strategies being considered for malaria elimination on an island close to the US mainland and its resources, and in an area with a relatively low level of malaria transmission.
